# Concurrent bevacizumab and temozolomide alter the patterns of failure in radiation treatment of glioblastoma multiforme

**DOI:** 10.1186/1748-717X-8-101

**Published:** 2013-04-25

**Authors:** Lisa BE Shields, Robert Kadner, Todd W Vitaz, Aaron C Spalding

**Affiliations:** 1 Norton Neuroscience Institute, Louisville, KY, USA; 2 The Brain Tumor Center, Norton Healthcare, Louisville, KY, USA; 3 DXP Imaging, Louisville, KY, USA; 4 The Norton Cancer Institute Radiation Center, Louisville, KY, USA; 5 Kosair Children’s Hospital, Louisville, KY, USA

**Keywords:** Glioblastoma multiforme, Radiation therapy, Bevacizumab, Temozolomide, Recurrence

## Abstract

**Background:**

We investigated the pattern of failure in glioblastoma multiforma (GBM) patients treated with concurrent radiation, bevacizumab (BEV), and temozolomide (TMZ). Previous studies demonstrated a predominantly in-field pattern of failure for GBM patients not treated with concurrent BEV.

**Methods:**

We reviewed the treatment of 23 patients with GBM who received 30 fractions of simultaneous integrated boost IMRT. PTV60 received 2 Gy daily to the tumor bed or residual tumor while PTV54 received 1.8 Gy daily to the surrounding edema. Concurrent TMZ (75 mg/m^2) daily and BEV (10 mg/kg every 2 weeks) were given during radiation therapy. One month after RT completion, adjuvant TMZ (150 mg/m^2 × 5 days) and BEV were delivered monthly until progression or 12 months total.

**Results:**

With a median follow-up of 12 months, the median disease-free and overall survival were not reached. Four patients discontinued therapy due to toxicity for the following reasons: bone marrow suppression (2), craniotomy wound infection (1), and pulmonary embolus (1). Five patients had grade 2 or 3 hypertension managed by oral medications. Of the 12 patients with tumor recurrence, 7 suffered distant failure with either subependymal (5/12; 41%) or deep white matter (2/12; 17%) spread detected on T2 FLAIR sequences. Five of 12 patients (41%) with a recurrence demonstrated evidence of GAD enhancement. The patterns of failure did not correlate with extent of resection or number of adjuvant cycles.

**Conclusions:**

Treatment of GBM patients with concurrent radiation, BEV, and TMZ was well tolerated in the current study. The majority of patients experienced an out-of-field pattern of failure with radiation, BEV, and TMZ which has not been previously reported. Further investigation is warranted to determine whether BEV alters the underlying tumor biology to improve survival. These data may indicate that the currently used clinical target volume thought to represent microscopic disease for radiation may not be appropriate in combination with TMZ and BEV.

## Introduction

Approximately 18,000 individuals are diagnosed annually with malignant primary brain tumors in the United States; more than half of these patients have GBM [1]. The current standard of care for newly diagnosed GBM is surgical resection followed by adjuvant radiotherapy with TMZ [2].

Radiation therapy following the initial surgical debulking procedure of the GBM represents one of the most effective postoperative treatment modalities. The volume of tumor treated has ranged from lower dose whole brain irradiation to higher dose focal tumor irradiation over the past thirty years in order to reduce the likelihood of normal tissue damage. Despite whole brain irradiation, most patients progress near or within the original tumor location. The primary goal in the treatment planning stage is to emphasize the dose delivery to the tumor volume while sparing the normal tissue [3,4].

Previous studies have demonstrated that the vast majority of patterns of failure following radiation were within the high dose in-field suggesting local tumor recurrence in close proximity to the primary tumor site [5-8]. Both BEV and TMZ have shown efficacy in conjunction with radiation in the treatment of recurrent GBMs [9-12], and BEV has been shown *in vitro* and in animal models to alter GBM cell migration [13]. Therefore, we investigated whether this intensification of therapy with biological agents altered the pattern of failure in patients with GBM.

## Methods

**Figure 1:**
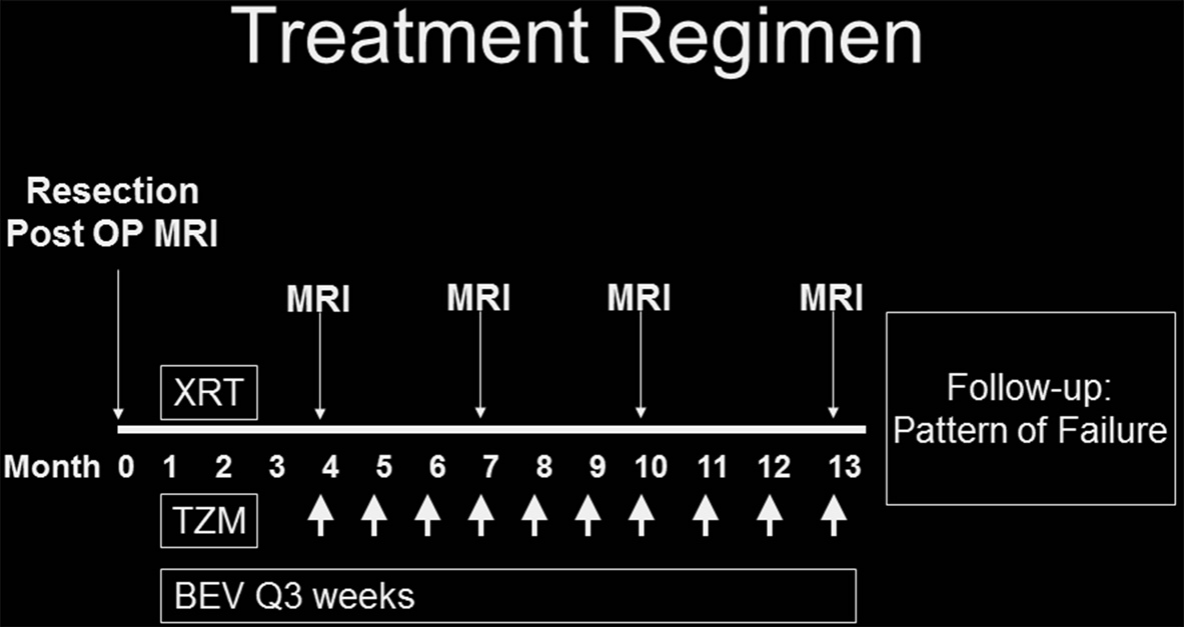
Treatment regimen of radiation therapy with BEV and TMZ for GBM.

Under an IRB-approved protocol and in compliance with the Helsinki Declaration, we retrospectively reviewed the treatment of 23 patients with newly diagnosed GBM who received post-operative 30 fractions of simultaneous integrated boost IMRT (Intensity-Modulated Radiation Therapy). The patients did not have any evidence of multifocal disease. The extent of resection (gross total, subtotal, or biopsy only) was determined on 24 hour postoperative MRI. Postoperative MRI scans were fused with the radiation planning CT scan. Targets were delineated according to RTOG guidelines with the GTV1 containing the postoperative T2 FLAIR abnormality, any postoperative enhancement, and the surgical cavity expanded 2.5 cm to generate the PTV60. GTV2 included the enhancing abnormality on the postoperative scan and surgical cavity and was expanded 1.5 cm to generate the PTV54. Concurrent TMZ (75 mg/m^2) daily and BEV (10 mg/kg every 2 weeks) were given during radiation therapy for 6 weeks. One month after completion of RT, adjuvant TMZ (150 mg/m^2 × 5 days) and BEV were delivered monthly until progression or 12 months total. An MRI scan with GAD was performed before initiation of adjuvant therapy and then every 3 months. Figure 1 depicts the treatment regimen.

Progression was defined as new T1 Gadolinium enhancement or T2 FLAIR progression, based on the RANO criteria [14]. The MRIs were independently reviewed by a neuroradiologist who was blinded to the previous radiation IDV (Isodose Volume). Recurrences were scored as in field (95% of recurrence volume in the 57 Gy, [95%] isodose volume), marginal (95% of the recurrence volume within the 48 Gy [80%] isodose volume), or distant (subepedymal versus deep white matter), consistent with prior reported studies [5,7,8]. In patients with recurrence based on progression of T2/FLAIR imaging, the diagnosis was made based on a combination of factors including mass effect, the development of new lesions, or the presence of progressive edema remote from the resection cavity. The time point of recurrence in these cases was based on the first sign of progressive disease, whether seen on FLAIR or T1 with Gadolinium. A biopsy was not performed due to the risk of complications as well as potential delay in additional anti-GBM therapy.

## Results

A total of 23 patients were enrolled in the study with an age range between 28 and 76 years (median age 55 years). Of the total 23 patients, the extent of resection of tumor was as follows: Gross total: 14 (61%), subtotal: 5 (22%), and biopsy only: 4 (17%). Table 1 summarizes the patient characteristics. Of the 10 patients who underwent methylation status testing, methylation of the MGMT promoter was observed in 5 of 10 patients (50%).

**Table 1:** Characteristics of patients with newly diagnosed GBM treated with radiation, BEV, and TMZ

**Characteristic**	**Number of patients (n = 23)**
Sex	
Male	15 (65%)
Female	8 (35%)
Age (years)	
Range	28–76
Median	55
Surgery	
Gross Total Resection	14 (61%)
Subtotal Resection	5 (22%)
Biopsy Only	4 (17%)
Number of BEV cycles	
Range	1–13
Median	4
Duration of Follow-Up (months)	
Range	10–67
Median	34
Status at Follow-Up	
Alive, without Progression	11 (48%)
Alive, with Progression of Disease	3 (13%)
Deceased from Disease	9 (39%)

With a median follow-up of 12 months, the median disease-free and overall survival was not reached. A total of 6 patients completed the full 12 cycles of adjuvant systemic therapy. The median number of cycles completed was four. Four patients discontinued therapy due to toxicity for the following reasons: bone marrow suppression (2), craniotomy wound infection (1), and pulmonary embolus (1). Three patients completed 3 or 4 cycles at the time of analysis without tumor progression or toxicity. Five patients had grade 2 or 3 hypertension that was managed by oral medications.

**Figure 2:**
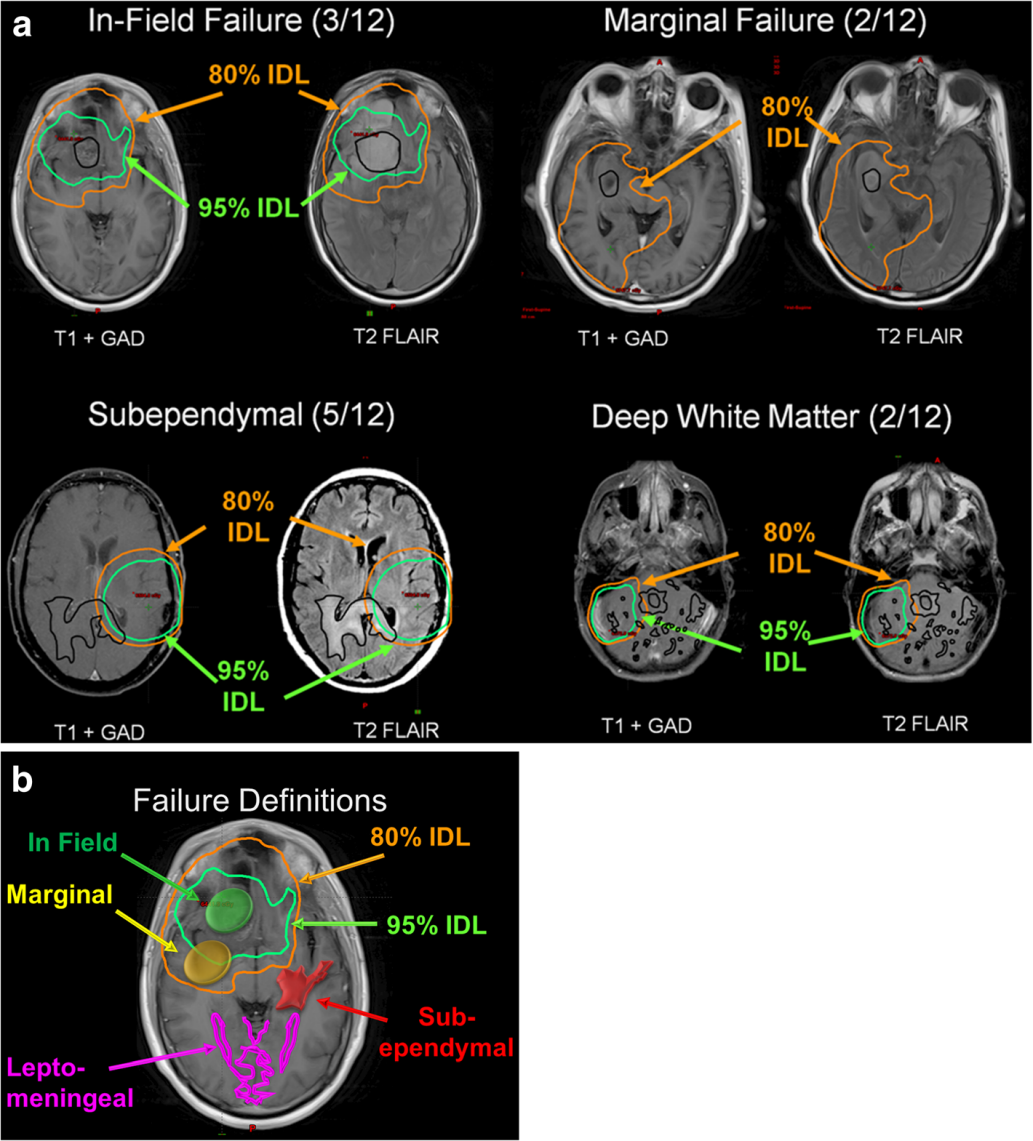
**(a, b) Patterns of failure following radiation therapy with BEV and TMZ for GBM.** Subependymal; Deep white matter; In-field failure (95% of recurrence volume in the 57 Gy, [95%] isodose volume); and Marginal failure (95% of the recurrence volume within the 48 Gy [80%] isodose volume).

Twelve of the 23 patients experienced a tumor recurrence (Figure 2a, b). Of the 12 patients with a recurrence, three patients (3/12; 25%) had in-field failure, and two (2/12; 17%) had marginal failure. Seven patients demonstrated distant failure with either subependymal (5/12; 41%) or deep white matter (2/12; 17%) spread detected on T2 FLAIR sequences. The pattern of recurrence on T2 was a combination of increased signal in the subependymal white matter, the corpus callosum, and in the fiber tracts of the corona radiata and centrum semiovale. The only leptomeningeal disease was that seen with GAD on T1. Table 2 highlights the patterns of failure with respect to number of cycles completed, the extent of resection, and the failure IDV. Five of twelve patients (41%) with a recurrence demonstrated evidence of GAD enhancement, specifically, in-field (3 of 5 patients) and marginal (2 of 5 patients). The pattern of failure observed in this study was statistically different with a P < 0.05 by ANOVA compared to published studies (Table 3).

**Table 2:** The patterns of failure following radiation for GBM with respect to cycles completed, extent of resection, and failure isodose volume

**Patient number**	**Pattern of failure**	**Cycles completed**	**Extent of resection**	**Failure IDV (Gy)**	**Failure IDV (%)**
**1**	**In-Field**	**7**	**GTR**	**58**	**96**
**2**	**In-Field**	**7**	**GTR**	**57**	**95**
**3**	**In-Field**	**12**	**GTR**	**58**	**97**
**4**	**Marginal**	**4**	**GTR**	**49**	**81**
**5**	**Marginal**	**5**	**STR**	**51**	**85**
**6**	**Subependymal**	**3**	**STR**	**24**	**40**
**7**	**Subependyma1**	**3**	**STR**	**12**	**20**
**8**	**Subependymal**	**3**	**Biopsy**	**21**	**35**
**9**	**Subependymal**	**3**	**STR**	**25**	**42**
**10**	**Subependymal**	**6**	**GTR**	**26**	**43**
**11**	**Deep White Matter**	**4**	**GTR**	**12**	**21**
**12**	**Deep White Matter**	**10**	**Biopsy**	**5**	**8**

GTR: Gross Total Resection.

STR: Subtotal Resection.

IDV: Isodose Volume.

**Table 3:** The patterns of failure following radiation for GBM with respect to radiation techniques, radiation dose, and percentage of in-field failure

**Study**	**Year**	**Number of patients**	**Radiation techniques**	**Dose**	**% In-field failure**
Current study	2013	23	IMRT	60 Gy	25% (p < .05)
Lee [22]	1999	36	3DCRT	70 or 80 Gy	89%
Hess [24]	1994	66	3DCRT	60 Gy	86%
Garden [7]	1991	39	3DCRT	65.4 Gy	71%
Wallner [8]	1989	25	Whole Brain + Boost	70 Gy	75%
Fitzek [6]	1999	23	Sequential Boost 3DCRT with photons and protons	90 CGE	78%
Chan [5]	2002	34	Sequential Boost 3DCRT	90 Gy	91%

Of the 12 patients who experienced a tumor recurrence, 10 had clinical symptoms which preceded the tumor progression detected on MRI. The symptoms included a change in mental status, new weakness of a limb, speech changes, and headaches. The tumor recurrences occurred between 132–560 days postoperatively (median: 256 days). The earliest recurrence occurred within 84 days postoperatively outside of the high dose IDV, which is not consistent with pseudoprogression. The tumor recurrence of two patients was noted on MRI prior to the initiation of symptoms 256 and 285 days postoperatively, respectively. However, both of these patients began experiencing symptoms within 3 weeks of imaging progression, and one died within one month of new symptoms.

## Discussion

Angiogenesis is necessary for the development of macroscopic neoplasia [15]. GBM exhibit a host of pathological features, including loss of blood-brain barrier integrity, endothelial proliferation, marked hypoxia, and tumor necrosis [11]. Attention has focused on developing antiangiogenic therapies that target either the vascular endothelial growth factor (VEGF) or vascular endothelial growth factor receptor (VEGFR) [9]. Several mechanisms of anti-VEGF agents in brain tumors have been proposed such as vascular normalization leading to improved tissue oxygenation and drug delivery, sensitization of endothelial cells to cytotoxic agents specifically radiation, and direct anti-glioma stem cell effect [9,11]. The most commonly observed side effects with BEV include hypertension, fatigue, and thrombosis with rare intratumor hemorrhage [9].

Both TMZ and BEV are recognized to provide clinical benefits for GBM patients [9-12]. TMZ is an alkylating agent which is rapidly absorbed and highly bioavailable after oral administration which simplifies patient dosing [10]. TMZ also crosses the blood-brain barrier to attain an effective concentration in the CNS [10]. The addition of TMZ to radiation has been shown in a randomized trial to improve survival in GBM patients [12]. BEV is an anti-VEGF monoclonal antibody which neutralizes VEGF, may decrease cerebral edema, and has been shown to improve survival in patients with progressive GBM compared with historical control [11,16]. BEV combined with cytotoxic chemotherapy (CPT-11 or carboplatin) have been shown to interrupt VEGF signaling causing sensitization or reversal of cytotoxic drug resistance, improvement in cytotoxic drug vascular access through vascular normalization, and a reduction in tumor interstitial pressure [9].

Based on these observations, investigators have studied the addition of BEV to the standard radiation and TMZ regimen. Lai and colleagues treated patients with external beam radiation of 60.0 Gy after the surgery concurrently with TMZ and BEV [17,18]. Upon completion of the radiation, patients continued to receive TMZ and BEV. They observed toxicities in their studies including radiation-induced optic neuropathy, retinal detachment, fatigue, myelotoxicty, wound breakdown, and deep venous thrombosis/pulmonary embolism [17,18]. Vredenburgh and colleagues investigated adding BEV to a combination of 59.4 Gy radiation and TMZ followed by a cocktail of BEV, TMZ, and irinotecan after the completion of the radiation [19,20]. Toxicities included thrombocytopenia, deep vein thrombosis/pulmonary embolism, gastrointestinal toxicity (bowel perforation, rectal abscess, and sepsis), fatigue, Pneumocysitis carinii pneumonia, and optic neuritis. While these side effects were noted in their patient population, Vredenburgh et al. reported the overall safety of adding BEV to radiation therapy and TMZ [19]. In Narayana et al.’s study, patients with a newly diagnosed GBM were treated with a combination of 59.4 Gy radiation, TMZ, and BEV [21]. Although a total of 20% of patients experienced Grade III/IV toxicities including thrombocytopenia, deep vein thrombosis, and pulmonary embolism, the authors reported that the addition of BEV to conventional therapy in the treatment of newly diagnosed GBM improved PFS and OS compared with historical controls [21].

The median follow-up of the present study was 12 months. While the median overall survival was not reached, the 6 month progression-free survival was promising (87%). In the current study, combined therapy of radiation in conjunction with BEV and TMZ to treat GBM proved successful with minimal toxicity. Four patients discontinued therapy due to toxicity for the following: bone marrow suspension (2), craniotomy wound infection (1), and pulmonary embolus (1). Five patients had grade 2 or 3 hypertension that was managed by oral medications.

The determination of tumor progression in this study was based on new T1 Gadolinium enhancement or T2 FLAIR progression, based on the RANO criteria [14]. A biopsy was not performed due to the risk of complications as well as potential delay in additional anti-GBM therapy. The risk of an unfavorable neurosurgical event outweighed the benefit of conducting a biopsy as prior BEV increases complications. Ten of the 12 patients who experienced a tumor recurrence had clinical symptoms prior to tumor progression detected on MRI. These new symptoms included a change in mental status, new weakness of a limb, speech changes, and headaches. Two of the 12 patients with a tumor recurrence denied any symptoms prior to the tumor detection on MRI. However, both of these patients began experiencing symptoms within 3 weeks of imaging progression, and one died within one month of new symptoms.

As the radiation treatment of GBM has evolved, investigators have analyzed the patterns of failure to determine whether the target volume margin was sufficient or whether it requires redefinition prior to subsequent trials [22]. In the 1970s the RTOG determined that the dose of 60 Gy was appropriate to administer to GBM patients [23]. As technology improved, investigators have steadily increased the radiation dose as repeated patterns of failure analysis have shown primarily in-field recurrences with the hypothesis that increased radiation will improve tumor control. Table 3 depicts the patterns of failure following radiation for high-grade gliomas in a host of studies in the literature with special attention to the radiation regimen and dose [5-8,22,24]. The majority (71%–91%) of failures were in-field. Three studies are of particular note within Table 3 due to dose escalation beyond 60 Gy. Fitzek et al. used sequential boost 3DCRT with photons and protons and a dose of 90 CGE to treat GBM patients [6]. The in-field failure was 78% with a median survival time of 20 months. Lee et al. utilized 3DCRT with either 70 or 80 Gy and demonstrated an 89% in-field failure [22]. They proposed dose escalation to 90 Gy and beyond while maintaining the same target volume definition criteria [22]. As a follow-up, Chan et al. used sequential boost 3DCRT with a dose escalation to 90 Gy and reported an in-field failure of 91% [5]. They suggested that intensification of local radiotherapy with dose escalation was feasible and warranted further evaluation [5]. Taken together, these studies suggest that radiation dose escalation alone is unlikely to produce clinical benefit for GBM patients.

Two recent trials have randomized patients to the standard radiation and TMZ regiment with or without BEV to help determine whether upfront BEV provides clinical benefit. The Avaglio phase III trial with BEV, TMZ, and RT in newly-diagnosed GBM has reached accrual, with initial results presented in abstract form [25]. The Avaglio study demonstrated improved PFS, however, the interim analysis of OS did not reach statistical significance [25]. The RTOG has completed accrual on a similar study, 0825, and results are pending.

In our current study, the most frequent failure location following radiation, BEV, and TMZ for GBM was distant (subependymal and deep white matter) with 7/12 patients (58%) while in-field failure was less common (3/12 patients, 25%). Without examining the brain tissue of a patient who experienced a tumor recurrence following treatment with radiation, BEV, and TMZ, it is difficult to determine the precise role that these biological agents play. BEV may have only blunted the ability of T1 + GAD to detect the recurrence of GBM and not actually eradicated the tumor in the in-field area. Future advances in the treatment of GBM with radiation, BEV, and TMZ will shed light on whether BEV affects the biological tumor composition to improve survival for patients with GBM. Pattern of failure analysis for patients on the Avaglio and RTOG 0825 studies may also further the understanding of this regimen.

## Conclusion

Combined modality therapy of radiation, BEV, and TMZ to treat GBM was well tolerated in the present study. Similar to other reports, BEV blunted the ability of T1 + GAD to detect recurrence of GBM. The predominant out-of-field patterns of failure in our cohort of radiation with the biological agents BEV and TMZ have not been previously reported. Additional follow-up is warranted to determine whether BEV alters the underlying tumor biology to improve survival or whether the imaging is modified. These data indicate that the currently used target expansions for radiation may not be appropriate in combination with TMZ and BEV.

## Consent

Written informed consent was obtained from the patients for publication of this report and any accompanying images.

## Competing interests

The authors declare that they have no competing interests.

## Authors’ contributions

LBES performed the literature search, analyzed the primary data, and played the primary role in the writing of the manuscript. RK interpreted all of the MRI done for the patients in this study and helped write the manuscript. TWV was the neurosurgeon who performed the GBM surgical extraction and analyzed the primary data. ACS was the radiation oncologist who designed the study, collected and analyzed the primary data, administered the radiation and chemotherapy to the patients, and wrote the manuscript. All authors read and approved the final manuscript.
